# Mutational spectrum of Chinese LGMD patients by targeted next-generation sequencing

**DOI:** 10.1371/journal.pone.0175343

**Published:** 2017-04-12

**Authors:** Meng Yu, Yiming Zheng, Suqin Jin, Qiang Gang, Qingqing Wang, Peng Yu, He Lv, Wei Zhang, Yun Yuan, Zhaoxia Wang

**Affiliations:** 1 Department of Neurology, Peking University First Hospital, Beijing, China; 2 Science and Technology, Precisionmdx Inc., Beijing, China; RIKEN Advanced Science Institute, JAPAN

## Abstract

This study aimed to study the diagnostic value of targeted next-generation sequencing (NGS) in limb-girdle muscular dystrophies (LGMDs), and investigate the mutational spectrum of Chinese LGMD patients. We performed targeted NGS covering 420 genes in 180 patients who were consecutively suspected of LGMDs and underwent muscle biopsies from January 2013 to May 2015. The association between genotype and myopathological profiles was analyzed in the genetically confirmed LGMD patients. With targeted NGS, one or more rare variants were detected in 138 patients, of whom 113 had causative mutations, 10 sporadic patients had one pathogenic heterozygous mutation related to a recessive pattern of LGMDs, and 15 had variants of uncertain significance. No disease-causing mutation was found in the remaining 42 patients. Combined with the myopathological findings, we achieved a positive genetic diagnostic rate as 68.3% (123/180). Totally 105 patients were diagnosed as LGMDs with genetic basis. Among these 105 patients, the most common subtypes were LGMD2B in 52 (49.5%), LGMD2A in 26 (24.8%) and LGMD 2D in eight (7.6%), followed by LGMD1B in seven (6.7%), LGMD1E in four (3.8%), LGMD2I in three (2.9%), and LGMD2E, 2F, 2H, 2K, 2L in one patient (1.0%), respectively. Although some characteristic pathological changes may suggest certain LGMD subtypes, both heterogeneous findings in a certain subtype and overlapping presentations among different subtypes were not uncommon. The application of NGS, together with thorough clinical and myopathological evaluation, can substantially improve the molecular diagnostic rate in LGMDs. Confirming the genetic diagnosis in LGMD patients can help improve our understanding of their myopathological changes.

## Introduction

Limb-girdle muscular dystrophy (LGMD) is a group of genetically heterogeneous disorders characterized by predominantly proximal muscle weakness and dystrophic features in pathology[1–4]. Based on the inheritance pattern, LGMDs are classified into autosomal dominant inherited LGMD1 and autosomal recessive inherited LGMD2[5, 6]. Currently, 32 LGMD subtypes of different genetic defects, including LGMD1A-1H and LGMD2A-2X, have been reported[4, 6]. According to the latest guidelines, the precise diagnoses of LGMDs rely on detailed clinical examination and muscle biopsy, as well as on genetic screening to detect the causative mutations[7]. Traditionally, patients suspected of LGMDs were screened for certain genes by Sanger sequencing based on their clinical and histopathological findings[3, 8], although Sanger sequencing and the gene-by-gene approach is neither economical nor efficient[9]. Recently, next-generation sequencing (NGS) has been widely applied in the fields of myology and other hereditary diseases, showing high efficiency and cost-effectiveness[9–14]. There have been several reports on the application of NGS as a first-tier test in the genetic screening of patients suspected of LGMDs. Still, the positive rate of identifying the causative mutation varied greatly, from 16% to 65%[15–19]. Additionally, different spectrums of LGMD subtypes in varying populations worldwide have been demonstrated [15–18]. Herein, we applied targeted NGS in a large cohort of Chinese patients suspected of LGMDs to evaluate the diagnostic rates of targeted NGS in LGMDs, and to investigate the relative frequencies of LGMD subtypes in China. The association between genotype and myopathological profiles was also analyzed.

## Methods

### Patient selection

**Fig 1:**
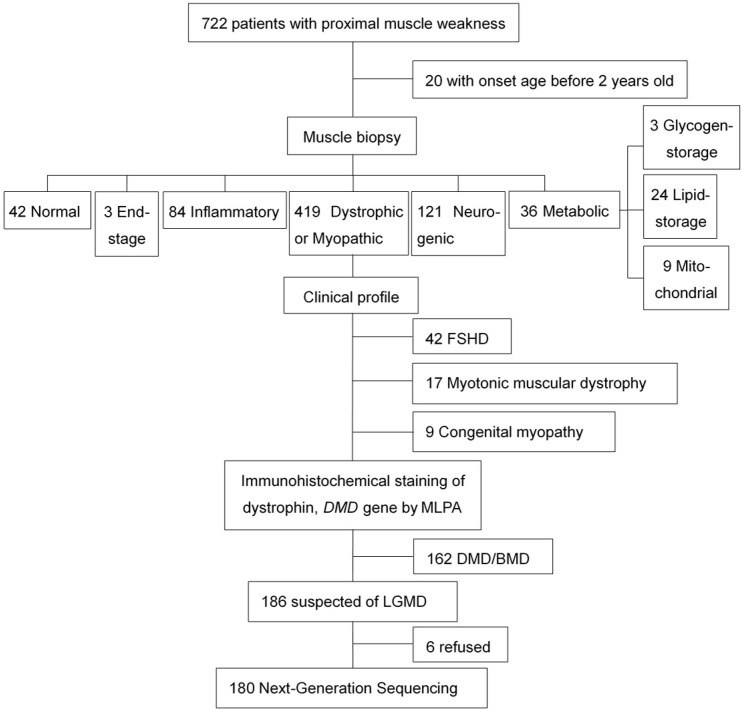
Flowchart of patients’ enrollment.

From January 2013 to May 2015, 1,872 patients presented with muscle weakness and were underwent muscle biopsy in the Department of Neurology, Peking University First Hospital, Beijing. As detailed in Fig 1, 180 patients who were highly suspected of LGMDs were recruited in this study according to the following inclusion and exclusion criteria:

Inclusion Criteria: 1. Onset age ≥ 2 years old. 2. Clinically presented with proximal muscle weakness, and muscle strength examination proved. 3. Muscle biopsy showed: (1) dystrophic or myopathic changes, i.e., the presence of degeneration and regeneration muscle fibers, with or without variation in fiber size, endomysial and perimysial fibrosis, and/or (2) immunohistochemical (IHC) staining or Western blotting showed decreased muscle related proteins. 4. Agreed to provide DNA samples for next-generation sequencing.

Exclusion Criteria: 1. Clinically, histopathologically, and genetically diagnosed facioscapulohumeral muscular dystrophy (FSHD), myotonic muscular dystrophy, or congenital myopathy. 2. Deletion/duplication of exons detected in the *DMD* gene using multiplex ligation-dependent probe amplification (MLPA) method. 3. Muscle biopsy and genetically confirmed mitochondrial myopathy, glycogen storage myopathy or lipid storage myopathy. 4. Muscle biopsy confirmed normal histological appearance without any specific pathological findings or end-stage histopathological appearance that muscle fibers were replaced by fibrous and fatty tissues.

The present study was approved by the Human Research Ethics Committee of Peking University First Hospital, and all participants provided written informed consent.

### Muscle biopsy

We performed muscle biopsy for all of the patients. Serial frozen sections were stained by routine histological and histochemical methods and by immunohistochemical methods for dystrophin (N-terminus, C-terminus, and rod domain, Novocastra), sarcoglycan complex, (α-, β-, and γ-, Novocastra), dysferlin (Chemicon), and desmin (Novocastra).

### Targeted next-generation sequencing

Genomic DNA samples were extracted from peripheral blood of 100 patients, and from muscle tissues of 80 patients. A neuromuscular disease panel (Agilent, USA) was designed to capture a 1.77 Mb region containing 6,242 exons (including the 10 bp flanking on either side) of 420 genes known to be associated with common inherited muscular diseases (S1 Table). Samples with index tag were pair-end sequenced simultaneously on Illumina HiSeq 2500 Sequencer (Illumina, San Diego, CA) for 150 cycles. FASTQ sequencing files were aligned with human genome assembly GRCh38 using Burrows-Wheeler Aligner[20], processed using Picard and jointly called with 2,000 control samples from an in-house Chinese cohort, using the GATK 3.0 Haplotype Caller. The data received had an average read depth of more than 200-fold coverage in more than 98% of the targeted regions.

### Identification of mutations and selection of candidate genes

First, variants with population frequency over 1% in the dbSNP v137, Exome Variant Server, 1000 Genome, and in-house Chinese database were filtered out. Second, only variants predicted to affect the coding regions (including non-synonymous, splice acceptor and donor site, and insertions or deletions [NS/SS/InDel]) were selected for further analysis. Third, variants were correlated with patients’ phenotypes, results of clinical investigations and myopathological changes. Finally, Sanger sequencing with specific primers was conducted to confirm the selected variants. When available, Sanger sequencing of the specific region in the gene was conducted in family members to testify the segregation.

## Results

### Clinical and pathological characteristics of enrolled patients

The 180 patients (105 male and 75 female) included in our cohort were from diverse districts of China (S1 Fig) and were unrelated. Based on family pedigrees, 25 patients (13.9%) had a positive family history. In total, 10 families had a likely autosomal dominant pattern of inheritance, 14 families were typical of an autosomal recessive pattern of inheritance, and one family was in accordance with X-linked inheritance. The median age of onset of the cohort was 17 (interquartile ranges 9 to 26) years old. The median value for maximum serum creatine kinase (CK) level was 2236 (interquartile ranges 539.25 to 5602.75) IU/L. All the enrolled patients received muscle biopsy; 128 patients (71.1%) showed dystrophic changes, while the rest 52 (28.9%) showed myopathic changes. Detailed information, including clinical characteristics, CK levels, and muscle biopsy findings, are shown in S2 Table and S3 Table.

### Genetic variants detected by targeted NGS

In 138 (76.7%) out of 180 patients tested, we detected one or more likely pathogenic variants in 28 different genes, and no pathogenic variants were found in 42 patients (23.3%) in any of the 420 myopathy-related genes (S4 Table). Of the 220 variants in different patients, we detected the following: 121 missense mutations, 25 nonsense mutations, 35 frameshift mutations, 3 non-frameshift mutations caused by small insertions or deletions, 3 gross insertions or deletions, and 33 splicing site mutations. The *DYSF* and *CAPN3* genes were most frequently seen in our cohort, accounting for 28.9% and 14.4% of the cases, respectively.

### Confirmation of genetic diagnoses with phenotypic characteristics

Together with clinical and pathological findings, variants were consistent with phenotypes in 113 patients (62.8%), and thus confirmed genetic diagnoses were achieved. Among them, 91 variants have been proved by family segregation, while DNA samples of family members in the rest 22 could not be acquired and thus segregation could not be confirmed. Among the 113 patients, LGMD related gene were detected in 95 patients, including 49 patients with *DYSF* mutations, 24 with *CAPN3* mutations, seven with *LMNA* mutations, five with *SGCA* mutations, four with *DES* mutations, three with *FKRP* mutations, one with *POMT1* mutations, one with *TRIM32* mutations, and one with *ANO5* mutations. In addition, the following was detected in the cohort and was considered pathogenic: nine patients had *DMD* gene point mutations, three had *GNE* mutations, two had *LAMA2* mutations, two had *COL6A2* mutations, one had *COL6A1* mutations, and one had *CLCN1* mutation.

There were 10 sporadic patients (5.6%) with only a single heterozygous variant, including two patients with *CAPN3* mutations, three with *DYSF* mutations, three with *SGCA* mutations, one with *SGCB* mutations, and 1 with *SGCD* mutations. The myopathological changes in these patients, especially the findings on immunohistochemical staining, strongly supported corresponding genotypes. Thus, the genetic diagnoses of these 10 patients were considered probable, and we achieved a positive genetic diagnosis in 68.3% (123/180) of this cohort.

Fifteen patients were found carrying variants predicted to be pathogenic but have not been reported associated with these patients’ phenotypes up to now. Therefore, these variants were thought to be of uncertain significance (VUS), which were detected in 12 genes: *MYOT*, *TTN* and *PLEC* related to LGMD; *COL6A1* and *ITGA7* related to congenital muscular dystrophy; *NEB*, *RYR1* and *KBTBD13* related to congenital myopathy; *FLNC* related to myofibrillar myopathy; *CHRNA1* related to congenital myasthenic syndrome; *MYH7* related to Laing distal myopathy; and *CLCN1* related to myotonia congenital (S5 Table).

### Mutational spectrum of LGMDs

**Fig 2:**
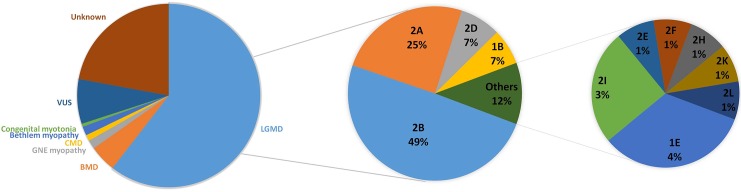
Overview of diagnoses of patients and spectrum of LGMD subtypes.

Totally, 105 patients were diagnosed as LGMDs clinically and genetically, including 95 patients with confirmed LGMD related gene mutations and 10 recessive LGMD patients, but only one causative mutation was found in the targeted-exon panel study. Among them, the top three subtypes were LGMD2B in 52 patients (49.5%, 17 homozygous, 32 compound heterozygous and three single heterozygous), LGMD2A patients in 26 (24.5%, five homozygous, 19 compound heterozygous and two single heterozygous), and LGMD 2D in eight patients (7.6%, five compound heterozygous and three single heterozygous). Subsequent to these subtypes were LGMD1B in seven patients (compound heterozygous), LGMD1E in four (compound heterozygous), LGMD2I in three (one homozygous and two compound heterozygous), LGMD2E in one (single heterozygous), LGMD2F in one (single heterozygous), LGMD2H in one (homozygous), LGMD2K in one (compound heterozygous), and LGMD2L in one (compound heterozygous) (Fig 2).

### Pathological findings based on confirmed molecular diagnoses

**Fig 3:**
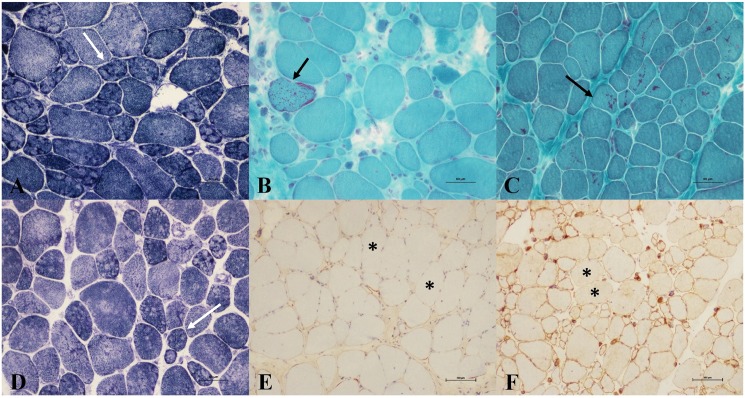
Myopathological changes of LGMD patients. A. NADH-TR staining shows lobulated fibers (arrow) in a LGMD2B patient. B. MGT staining shows red-ragged fibers (arrow) in a LGMD2B patient. C. MGT staining shows nemaline bodies (arrow) in a LGMD2A patient. D. NADH-TR staining shows multi-minicores (arrow) in a LGMD2A patient. E. Immunohistochemical labelling of N-terminal dystrophin shows decreased or absent expression (asterisk) in a LGMD2A patient. F. Immunohistochemical labelling of dysferlin shows decreased expression (asterisk) in a LGMD2A patient. Abbreviations: LGMD = limb-girdle muscular dystrophy; NADH-TR = nicotinamide adenine dinucleotide-tetrazolium reductase; MGT = modified Gomori trichrome.

During routine histopathological examinations, several specific abnormalities were detected (Table 1, Fig 3). Lobulated fibers were observed in nine LGMD2A patients, nine LGMD2B patients, one LGMD1B patient, one LGMD2D patient, and one LGMD2L patient. Nemaline bodies and multi-minicores were found in two LGMD2B patients. Features of abnormal mitochondria, including presence of ragged-red fibers (RRFs), ragged-blue fibers (RBFs), and cytochrome *c* oxidase (COX) deficient fibers were observed in 19 LGMD2B patients, two LGMD2A patients, one LGMD1E patient and one LGMD2E patient.

**Table 1:** Histopathological abnormalities in different LGMD subtypes^a^.

	Lobulated Fibers	Nemaline bodies	Multi-minicores	Abnormal Mitochondria (RRFs, RBFs or COX-)
LGMD1B	1/7			
LGMD1E				1/4
LGMD2A	9/26	2/26	2/26	2/26
LGMD2B	9/52			19/52
LGMD2D	1/8			
LGMD2E				1/1
LGMD2H				
LGMD2L	1/1			

^a^ Patients with LGMD2F, LGMD2I or LGMD2K were not presented with listed histopathological abnormalities.

Abbreviations: LGMD = limb-girdle muscular dystrophy; RRF = ragged-red fiber; RBF = ragged-blue fiber; COX- = cytochrome *c* oxidase deficient fiber.

By immunohistochemical analysis, abnormal expressions of myofibril membrane proteins (dystrophin, dysferlin, sarcoglycan complex) were observed (Table 2, Fig 3). Absent or decreased dystrophin expression was found in three LGMD1B patients, one LGMD1E patient, 11 LGMD2A patient, eight LGMD2B patients, five LGMD2C-2F patients and one LGMD2L patient. Absent or decreased dysferlin expression was observed in two LGMD2A and all 52 LGMD2B patients. Absent or decreased sarcoglycan complex expression was observed in two LGMD1B patients, six LGMD2A patients, five LGMD2B patients, all 10 LGMD2C-2F patients and one LGMD2I patient.

**Table 2:** Immunohistochemical findings in different LGMD subtypes^a^.

	Dystrophin ^b^	Dysferlin ^b^	Sarcoglycan complex ^b^
LGMD1B	3/7		2/7
LGMD1E	1/4		
LGMD2A	11/26	2/26	6/26
LGMD2B	8/52	52/52	5/52
LGMD2C-2F	5/10		10/10
LGMD2I			1/3
LGMD2K			
LGMD2L	1/1		

^a^ Patients with LGMD2H were not presented with listed histopathological findings.

^b^ Absent or decreased expression of dystrophin, dysferlin or sarcoglycan complex.

Abbreviations: LGMD = limb-girdle muscular dystrophy.

## Discussion

We used targeted next-generation sequencing to evaluate the mutational spectrum and prevalence of LGMDs in a Chinese population in a medical neuromuscular center in China. The enrollment of suspected LGMD patients was based on clinical manifestations and myopathological changes. According to previous studies, LGMDs are defined for those with proximal limb weakness with dystrophic or myopathic changes on muscle biopsy. [15] Certain subtypes of LGMDs could show non-specific changes or even normal on muscle biopsy,[7] which may lead to underestimate specific subtypes in our study. However, non-specific myopathological changes are mainly reported in specific LGMD1 subtypes,[21] which were occasionally reported in previous epidemiological researches,[22, 23] thus would minimally affect the results of our study.

There have been several studies on the diagnosis of LGMDs using NGS[15–18]. Most studies have focused on the diagnostic value of targeted NGS in clinically or pathologically suspected LGMD patients, and the average diagnostic positive rate was approximately 30%[16–18, 24]. Other studies used NGS as a second-tier diagnostic approach in patients undiagnosed by Sanger sequencing and protein analyses, which resulted in an extra diagnostic rate of 16%-45%[15, 19, 25]. The relatively high diagnostic rate in our cohort was partly due to detailed phenotyping on both clinical characteristics and myopathological findings. Meanwhile, the rapid development of NGS technology allowed a larger gene capture chip[26], which also contributed to the increased diagnostic rate. However, diagnoses of several subtypes of LGMD, including LGMD2B and LGMD2C-2F, could be made based on immunohistochemical findings, thus greatly improving the diagnostic rate in this study. Therefore, the diagnostic rate could be widely different depending on the inclusion criteria of patients in different studies.

In the present study, we have diagnosed 11 different LGMD subtypes by clinical, histological and targeted NGS analyses, and determined the frequency of different LGMD subtypes based in a Chinese population. Our cohort is the largest Chinese LGMD cohort reported thus far. All our patients were recruited consecutively in a given time period and were from various parts of China; therefore, the likelihood is that the sample generally reflects the Chinese LGMD population and the relative frequency of LGMD subtypes in China. Our research was different from a prior study in northeastern China, in which the most common subtypes were LGMD2A, 2B, 2D and 1C successively by muscle biopsy without proved genetic diagnosis[27]. Several other studies have reported different distributions of LGMDs subtypes in different countries or populations. The most common subtypes were LGMD 2A (8%) and LGMD2B (5%) in Australia,[28] dystroglycanopathy and LGMD 2A in the United States,[29] LGMD2I in Germany and Finland,[30] and LGMD2B, LGMD1B, 2A and 2G in Korea.[17] The spectrum difference between populations may be due to the disparate ethnic genetics and the effect of environmental factors. Differences in cohort size, study design, and strategies of gene examination may also influence the results.

In our study, the number of LGMD2B patients was the largest LGMD subtype in China and was underestimated by previous research. All 52 LGMD2B patients presented with decreased or absent expression of dysferlin on immunohistochemical staining, which addressed the significance of combining muscle biopsy and gene sequencing in order to avoid missing diagnoses of LGMD2B. Interestingly, LGMD2B patients also constituted the largest part of LGMDs in a study from Korean patients, and the percentages were also similar (49.5% in our study vs. 57.1% in Korean patients)[17]. In both Chinese and Korean patients with dysferlinopathy, the mutations spanned the whole length of *DYSF* gene, although there was a trend that mutations clustered in the N-terminal region of *DYSF* gene (S4 Table), suggesting a possible common mutation hot spot in China and Korea[31, 32]. However, both the present study and our previous research failed to reveal any geographical cluster mutation in Chinese patients with dysferlinopathy (data not shown)[31]. And we found no genotype-phenotype correlation between the site of mutation or its type, with the onset age and other clinical phenotype, which is consistent with other studies (S2 and S4 Tables)[31, 33]. Notably, pathological evidence of abnormal mitochondrial functions was frequently observed in our cohort patients with LGMD2B. Both pathological and biochemical evidence of mitochondrial dysfunction had also been reported in other studies[34, 35]. The possible mechanism of mitochondrial aberrations has been proposed due to respiratory chain deficiency caused by an increased cytosolic Ca^2+^ concentration[35].

Lobulated fibers have been reported with high prevalence in LGMD2A patients[36], but was present in only 34.6% of our LGMD2A patients, compared with 66.7% in another report on Chinese calpainopathy patients[36]. Meanwhile, we also identified lobulated fibers in 13 patients with other diagnoses. Previous studies have reported lobulated fibers were more frequent in patients with 550delA mutation in *CAPN3* [37], but none of the nine patients with lobulated fibers in our cohort shared the same mutation. Additionally, a variety of histopathological abnormalities were detected in LGMD2A patients, including nemaline bodies and multi-minicores, indicating lobulated fibers were neither a sensitive nor a specific sign in LGMD2A. On immunohistochemical staining, absent or decreased expression of dystrophin was observed in 42.3% of the LGMD2A patients. This could be mistaken for dystrophinopathy, without genetic confirmation. Furthermore, decreased expressions of dysferlin or sarcoglycan complex were detected in LGMD2A patients. Therefore, the histopathological and immunohistochemical manifestations of muscle biopsy in LGMD2A patients could be rather heterogeneous. Genetic sequencing, especially next-generation sequencing, could play an effective role in the diagnosis of LGMD2A.

Using targeted next-generation sequencing, we also confirmed seven BMD patients and two female carriers of with point mutations in the *DMD* gene and were undetected by MLPA methods. All patients showed absent or decreased expression of dystrophin on immunohistochemical staining, proving the effectiveness of NGS combined with MLPA in the diagnoses of dystrophinopathies[38, 39].

Notably, only one pathological variant was found in 10 sporadic patients with recessive pattern of LGMDs, including two LGMD2A patients, three LGMD2B patients, three LGMD2D patients, one LGMD2E patient and one LGMD2F patient. However, the clinical profiles, including muscle MRI findings and muscle biopsy results, were all in accordance with the suggested diagnoses. Hence, there may be other pathogenic variants which were not detected by targeted NGS, including variants in the promotor regions, gross insertions/ deletions, etc. Similarly, single heterozygous mutation with a recessive inheritance pattern was also reported in other assessments[16, 17]. This indicates that NGS has shortcomings in the diagnoses of LGMDs, and should be complemented by other technologies. However, a dominantly inherited LGMD case caused by a heterozygous mutation in the CAPN3 was also reported[40], which further challenges the analysis of the pathogenicity of variants detected by NGS.

Of the 42 patients with no variants detected by targeted NGS, muscle biopsy showed dystrophic changes in 23 patients, myopathic changes with rimmed vacuoles in four patients, and myopathic changes in 15 patients. Five of the patients had family histories highly suggestive of autosomal recessive pattern, and four had an autosomal dominant pattern. Whole-exome or whole-genome sequencing could be of high value in detecting potential pathological variants in these patients[15].

According to our study, targeted NGS is more cost and time efficient than the conventional strategies in LGMDs by Sanger sequencing of suspected targeted genes as the first-tier approach. Due to the overlapping clinical and pathological manifestations, as well as expanding phenotype spectrums, it has been increasingly challenging even for experienced specialists to choose the appropriate targeted gene. For instance, different subtypes of sarcoglycanopathies (LGMD2C-2F) show no distinguishing features clinically, and the absent or decreased expressions of different sarcolemmal components often coexist which can hamper precise diagnoses[41]. Under such conditions, targeted NGS shows its advantages compared with Sanger sequencing of every single gene. It is also noteworthy that gross deletions or duplications were implied by NGS and confirmed by MLPA in three patients in our cohort using data-based algorithms[42] and would have been missed by traditional Sanger sequencing.

### Conclusion

LGMD 2B and LGMD 2A were the most common LGMD subtypes in our China-based cohort. The combination of thorough clinical and myopathological evaluation with the advanced technique of next generation sequencing can greatly help improve the molecular diagnostic success rate in LGMD. We believe NGS could be considered as the first-tier test in patients suspected of LGMDs with supporting evidence including positive family history and accordant myopathological changes. Furthermore, confirming the genetic diagnosis in LGMD patients can help improve our knowledge of their myopathological changes.

## Supporting information

S1 Table 420 genes included in the neuromuscular disease panel. (DOCX)

S2 Table Clinical features of 180 patients suspected of LGMD. (DOCX)

S3 Table Myopathological features of 180 patients suspected of LGMD. (DOCX)

S4 Table Variants detected by targeted NGS in 180 patients suspected of LGMD. (DOCX)

S5 Table Clinical and pathological features of 15 patients with variants of uncertain significance. (DOCX)

S1 Fig Geographic distribution of included patients. (TIFF)
