# Supplementary material for: Mutational spectrum of Chinese LGMD patients by targeted next-generation sequencing
Source: PLoS One. 2017 Apr 12;12(4):e0175343. doi: 10.1371/journal.pone.0175343 (PMC5389788; doi:10.1371/journal.pone.0175343)
Supplement: S2 Table — (DOCX) [file pone.0175343.s002.docx]

**Table e-2 Clinical features of 180 patients suspected of LGMD**

| No | Sex | Age | Onset Age | Severity^a^ | Distribution^b^ | Distal | Neck flexor | Extra-ocular | Facial | Hypertrophy | Contracture | Myalgia | Dysphagia | Dysarthria | Winging scapula | Spinal deformity | Cardiac | Respiratory | Intelligence | Audition | | CK^c^ | |
| --- | --- | --- | --- | --- | --- | --- | --- | --- | --- | --- | --- | --- | --- | --- | --- | --- | --- | --- | --- | --- | --- | --- | --- |
| 1 | F | 20 | 13 | O | L | + | + |  |  |  | + |  |  |  | + |  |  |  |  |  | 350 | |  |
| 2 | M | 30 | 23 | M | E | + |  |  |  |  |  | + |  |  |  |  |  |  |  |  | 13400 | |  |
| 3 | F | 14 | 2 | S | E | + | + |  | + |  | + |  |  |  |  |  |  |  |  |  | 160 | |  |
| 4 | M | 20 | 16 | O | L | + | + |  |  |  |  |  |  |  |  |  |  |  | + |  | 175 | |  |
| 5 | M | 5 | 2 | S | E |  | + |  |  |  |  |  |  |  |  |  |  |  |  |  | 224 | |  |
| 6 | M | 24 | 20 | O | L |  |  |  |  | + |  |  |  |  | + |  |  |  |  |  | 4000 | |  |
| 7 | M | 14 | 14 | O | E |  |  |  |  |  |  | + |  |  |  |  | + |  |  |  | 9344 | |  |
| 8 | F | 30 | 21 | O | L |  |  |  |  |  |  | + |  |  |  |  |  |  |  |  | 4000 | |  |
| 9 | F | 42 | 39 | O | L | + |  |  |  |  |  |  |  |  |  |  |  |  |  |  | 492 | |  |
| 10 | M | 4 | 2 | S | E | + |  |  |  |  |  |  | + | + |  |  |  |  | + |  | 1038 | |  |
| 11 | M | 47 | 37 | O | L |  |  |  |  |  |  |  |  |  |  |  | + |  |  |  | 1064 | |  |
| 12 | M | 4 | 3 | S | E |  | + |  |  | + | + | + |  |  |  |  |  |  |  |  | 1300 | |  |
| 13 | M | 16 | 2 | O | E | + | + |  |  |  | + |  |  |  |  | + |  |  |  |  | 346 | |  |
| 14 | F | 26 | 25 | S | L | + |  |  |  |  |  |  |  |  | + |  |  |  |  |  | 514 | |  |
| 15 | F | 28 | 10 | S | L | + | + |  |  |  | + |  |  |  |  |  |  |  |  |  | 2300 | |  |
| 16 | F | 18 | 17 | M | L |  |  |  |  |  |  | + |  |  |  |  |  |  |  |  | 5065 | |  |
| 17 | M | 21 | 17 | M | L | + |  |  |  |  |  |  |  |  |  |  |  |  |  |  | 12059 | |  |
| 18 | M | 35 | 32 | M | L | + |  |  |  |  |  |  |  |  |  |  |  |  |  |  | 5833 | |  |
| 19 | M | 20 | 15 | O | L |  |  | + | + |  |  | + | + | + |  |  |  |  | + |  | 804 | |  |
| 20 | F | 51 | 45 | M | L | + |  |  |  |  |  |  |  |  |  |  |  |  |  |  | 2200 | |  |
| 21 | F | 9 | 5 | O | L |  | + |  |  | + |  |  |  |  |  |  |  |  |  |  | 10300 | |  |
| 22 | F | 49 | 29 | S | L | + | + |  |  |  |  |  |  |  |  |  |  |  |  |  | 1710 | |  |
| 23 | M | 64 | 46 | S | L | + | + |  |  |  |  |  |  |  |  |  |  |  |  |  | 413 | |  |
| 24 | M | 26 | 12 | S | L | + | + |  |  |  |  | + |  |  |  |  |  |  |  |  | 521 | |  |
| 25 | M | 28 | 22 | O | L | + | + |  |  |  |  |  |  |  |  |  |  |  |  |  | 5271 | |  |
| 26 | M | 22 | 5 | O | L | + |  |  |  |  |  |  |  |  |  |  |  |  |  |  | 557 | |  |
| 27 | M | 5 | 3 | O | U |  |  |  |  |  |  |  |  |  | + |  |  |  |  |  | 213 | |  |
| 28 | M | 20 | 20 | M | L |  |  |  |  |  |  |  |  |  |  |  |  |  |  |  | 12187 | |  |
| 29 | M | 36 | 26 | O | L | + |  |  |  |  |  |  |  |  |  |  |  |  |  |  | 2727 | |  |
| 30 | M | 45 | 31 | O | L |  | + |  |  |  |  |  |  |  |  |  |  |  |  |  | 314 | |  |
| 31 | F | 53 | 23 | O | L | + | + |  |  |  |  |  |  |  |  |  |  |  |  |  | 367 | |  |
| 32 | M | 36 | 25 | O | L | + |  |  |  |  |  |  |  |  |  |  |  |  |  |  | 4859 | |  |
| 33 | M | 23 | 20 | M | L | + |  |  |  |  |  |  |  |  |  |  |  |  |  |  | 12404 | |  |
| 34 | F | 49 | 46 | S | L |  | + |  |  |  |  |  |  |  |  |  |  |  |  |  | 680 | |  |
| 35 | M | 19 | 12 | S | L | + |  |  |  |  | + |  |  |  |  | + |  |  |  |  | 977 | |  |
| 36 | M | 34 | 29 | S | L | + |  |  |  |  |  |  |  |  |  |  |  |  |  |  | 3367 | |  |
| 37 | M | 57 | 50 | M | L | + |  |  |  |  |  |  |  |  |  |  |  |  |  |  | 1000 | |  |
| 38 | F | 35 | 29 | O | U |  | + |  |  |  |  |  |  |  |  |  |  |  |  |  | 542 | |  |
| 39 | F | 37 | 26 | S | L | + | + |  |  |  |  |  |  |  |  |  |  |  |  |  | 2887 | |  |
| 40 | F | 20 | 18 | M | L | + |  |  |  |  | + |  |  |  |  |  |  |  |  |  | 261 | |  |
| 41 | M | 22 | 18 | M | L | + |  |  |  |  |  | + |  |  |  |  |  |  |  |  | 2202 | |  |
| 42 | M | 15 | 2 | O | U | + |  |  |  | + | + |  |  |  | + |  |  |  |  |  | 653 | |  |
| 43 | M | 13 | 9 | O | L |  |  |  |  |  |  |  |  |  |  |  | + |  |  |  | 7800 | |  |
| 44 | M | 13 | 2 | S | U | + |  |  |  |  |  |  |  |  |  |  |  |  |  |  | 38 | |  |
| 45 | F | 14 | 2 | S | L |  | + |  |  |  |  |  |  |  |  |  | + |  |  |  | 521 | |  |
| 46 | M | 28 | 18 | O | L | + |  |  |  |  |  |  |  |  |  | + |  |  |  |  | 4000 | |  |
| 47 | F | 25 | 16 | M | L | + |  |  |  |  | + |  |  |  |  |  |  |  |  |  | 33320 | |  |
| 48 | M | 32 | 26 | M | L | + |  |  |  |  |  |  |  |  |  |  |  |  |  |  | 7377 | |  |
| 49 | M | 18 | 2 | O | L | + |  |  |  |  | + |  |  |  |  |  |  |  |  |  | 929 | |  |
| 50 | F | 32 | 26 | M | L |  |  |  |  |  |  |  |  |  |  |  |  |  |  |  | 4300 | |  |
| 51 | F | 38 | 28 | O | L | + | + |  |  |  |  |  |  |  |  |  |  |  |  |  | 3000 | |  |
| 52 | F | 32 | 12 | M | L |  |  |  |  |  | + |  |  |  |  |  |  |  |  |  | 34 | |  |
| 53 | F | 12 | 2 | M | L | + | + |  |  |  |  |  |  |  |  |  |  |  |  |  | 1555 | |  |
| 54 | F | 9 | 3 | O | E |  | + |  |  |  |  |  |  |  |  |  |  |  |  |  | 216 | |  |
| 55 | F | 3 | 2 | S | L |  | + |  |  |  |  |  |  |  |  |  |  |  |  |  | 2623 | |  |
| 56 | M | 24 | 19 | O | L | + |  |  |  |  |  |  |  |  |  |  |  |  |  |  | 6730 | |  |
| 57 | M | 14 | 14 | M | L |  |  |  |  |  | + | + |  |  |  |  |  |  |  |  | 8955 | |  |
| 58 | M | 26 | 23 | M | L |  |  |  |  | + |  |  |  |  |  |  |  |  |  |  | 3119 | |  |
| 59 | M | 22 | 12 | M | L |  | + |  |  |  |  |  |  |  |  |  |  |  |  |  | 6844 | |  |
| 60 | M | 5 | 2 | M | L |  | + |  |  |  |  |  |  |  |  |  |  |  |  |  | 1000 | |  |
| 61 | M | 7 | 4 | M | L | + | + |  |  |  | + |  |  |  |  |  |  |  |  |  | 831 | |  |
| 62 | F | 17 | 7 | M | L |  |  |  |  | + | + |  |  |  | + | + |  |  |  |  | 3351 | |  |
| 63 | M | 14 | 2 | M | L | + |  |  |  | + | + | + |  |  |  |  | + |  |  | + | 10000 | |  |
| 64 | M | 29 | 19 | M | L | + |  |  |  |  |  | + |  |  |  |  |  |  |  |  | 397 | |  |
| 65 | F | 41 | 24 | S | L | + |  |  |  |  |  | + |  |  |  |  |  |  |  |  | 370 | |  |
| 66 | F | 25 | 13 | S | L | + | + |  |  |  | + |  |  |  |  | + |  |  |  |  | 216 | |  |
| 67 | M | 6 | 2 | M | E | + | + |  |  |  | + |  |  |  |  |  |  |  |  |  | 568 | |  |
| 68 | M | 66 | 31 | O | L |  |  |  |  |  |  |  |  |  | + | + |  |  |  |  | 290 | |  |
| 69 | M | 43 | 8 | S | L | + |  |  |  | + |  |  |  |  | + |  |  |  |  |  | 1190 | |  |
| 70 | F | 24 | 14 | S | L | + |  |  |  |  |  |  |  |  |  |  |  |  |  |  | 7000 | |  |
| 71 | F | 25 | 22 | M | L |  |  |  |  |  |  |  |  |  |  |  |  |  |  |  | 12479 | |  |
| 72 | M | 39 | 22 | S | U | + |  |  |  |  |  |  |  |  |  |  |  |  |  |  | 438 | |  |
| 73 | F | 33 | 23 | O | L |  |  |  |  |  |  |  |  |  | + |  |  |  |  |  | 5453 | |  |
| 74 | F | 14 | 14 | M | L |  | + |  |  |  |  |  |  |  |  |  |  |  |  |  | 8000 | |  |
| 75 | F | 24 | 22 | M | L | + |  |  |  |  |  |  |  |  |  |  |  |  |  |  | 152 | |  |
| 76 | M | 27 | 3 | M | L |  |  |  |  |  |  |  |  |  |  |  |  |  |  |  | 5371 | |  |
| 77 | M | 37 | 18 | S | L | + | + |  |  |  |  |  |  |  |  |  |  |  |  |  | 4922 | |  |
| 78 | F | 28 | 12 | S | E | + | + |  |  |  |  |  |  |  |  |  |  |  |  |  | 763 | |  |
| 79 | M | 19 | 19 | M | L |  |  |  |  |  |  |  |  |  |  |  |  |  |  |  | 214 | |  |
| 80 | M | 15 | 14 | M | L |  |  |  |  |  |  |  |  |  |  |  |  |  |  |  | 31841 | |  |
| 81 | M | 14 | 2 | M | L |  |  |  |  |  |  |  |  |  |  |  |  |  |  |  | 1200 | |  |
| 82 | F | 18 | 3 | O | L | + |  |  |  |  |  |  |  |  |  |  |  |  |  |  | 2997 | |  |
| 83 | F | 49 | 42 | S | U |  |  |  |  |  |  |  |  |  |  |  |  |  |  |  | 2191 | |  |
| 84 | M | 49 | 43 | O | L | + | + | + |  |  |  |  |  |  | + |  |  |  |  |  | 487 | |  |
| 85 | M | 12 | 5 | M | L |  |  |  |  |  |  | + |  |  |  |  |  |  |  |  | 2331 | |  |
| 86 | M | 38 | 28 | S | L | + |  |  |  |  |  |  |  |  |  |  |  |  |  |  | 338 | |  |
| 87 | M | 24 | 20 | O | L | + |  |  |  |  |  |  |  |  |  |  | + |  |  |  | 15000 | |  |
| 88 | F | 23 | 12 | O | E | + |  |  |  | + |  |  |  |  |  |  |  |  |  |  | 5118 | |  |
| 89 | F | 10 | 2 | O | E | + |  |  |  |  | + |  |  |  |  |  |  |  |  |  | 17690 | |  |
| 90 | M | 24 | 10 | M | L | + |  |  |  | + |  |  |  |  |  |  |  |  |  |  | 4234 | |  |
| 91 | M | 26 | 22 | M | E | + |  |  |  |  |  |  |  |  |  |  |  |  |  |  | 162 | |  |
| 92 | M | 7 | 2 | S | L | + | + |  |  |  | + |  |  |  |  |  |  |  |  |  | 1358 | |  |
| 93 | F | 13 | 7 | O | L |  | + |  |  |  |  |  |  |  |  |  |  |  |  |  | 93 | |  |
| 94 | M | 41 | 26 | O | L |  |  |  |  |  |  |  |  |  |  |  |  |  |  |  | 123 | |  |
| 95 | M | 14 | 12 | M | E | + |  |  |  |  | + |  |  |  |  |  |  |  |  |  | 12776 | |  |
| 96 | F | 28 | 20 | O | L | + |  |  |  |  |  | + |  |  |  |  |  |  |  |  | 4548 | |  |
| 97 | F | 53 | 43 | S | L | + | + |  |  |  | + |  |  |  |  |  |  |  |  |  | 214 | |  |
| 98 | M | 29 | 25 | M | L | + |  |  |  |  |  |  |  |  |  |  |  |  |  |  | 8121 | |  |
| 99 | M | 18 | 15 | M | L |  |  |  |  |  |  | + |  |  |  |  |  |  |  |  | 5000 | |  |
| 100 | F | 3 | 3 | S | L | + | + |  |  |  |  |  |  |  |  |  |  |  |  |  | 9560 | |  |
| 101 | F | 37 | 32 | O | L |  | + |  |  |  |  |  |  |  | + |  |  |  |  |  | 1274 | |  |
| 102 | M | 5 | 5 | M | L |  |  |  |  |  |  |  |  |  |  |  |  |  |  |  | 6069 | |  |
| 103 | M | 16 | 15 | M | L | + |  |  |  |  |  |  |  |  |  |  |  |  |  |  | 543 | |  |
| 104 | M | 40 | 29 | M | L |  |  |  |  |  |  | + |  |  |  |  |  |  |  |  | 2164 | |  |
| 105 | M | 19 | 16 | M | L | + |  |  |  |  | + |  |  |  |  |  |  |  |  |  | 269 | |  |
| 106 | M | 35 | 25 | O | L | + |  |  |  |  |  |  |  |  |  |  | + |  |  |  | 843 | |  |
| 107 | M | 14 | 2 | O | E | + |  |  |  |  |  |  |  |  |  | + | + |  |  |  | 173 | |  |
| 108 | M | 34 | 24 | M | U |  | + |  |  |  |  |  |  |  |  |  |  |  |  |  | 4327 | |  |
| 109 | F | 4 | 4 | O | L | + | + |  |  |  |  | + |  |  |  |  |  |  |  |  | 7456 | |  |
| 110 | M | 45 | 44 | M | L |  |  |  |  |  |  | + |  |  |  |  |  |  |  |  | 1225 | |  |
| 111 | F | 49 | 29 | O | L |  |  |  |  | + |  |  |  |  |  |  | + |  |  |  | 2055 | |  |
| 112 | M | 26 | 23 | S | L | + | + |  |  |  |  |  |  |  |  |  |  |  |  |  | 997 | |  |
| 113 | F | 25 | 24 | O | L | + |  |  |  |  |  |  |  |  |  |  |  |  |  |  | 10483 | |  |
| 114 | M | 13 | 10 | M | L |  |  |  |  |  |  | + |  |  |  |  | + |  |  |  | 7001 | |  |
| 115 | F | 18 | 13 | M | L |  |  |  |  |  |  |  |  |  |  |  |  |  |  |  | 8014 | |  |
| 116 | M | 17 | 9 | S | L | + |  |  |  |  | + |  |  |  |  |  |  |  |  |  | 7471 | |  |
| 117 | F | 19 | 17 | O | L |  | + |  |  |  |  |  |  |  |  |  |  |  |  |  | 2100 | |  |
| 118 | M | 22 | 15 | O | L | + |  |  |  |  |  |  |  |  | + |  |  |  |  |  | 1009 | |  |
| 119 | F | 33 | 7 | S | E | + |  |  |  |  |  |  |  |  |  |  |  |  |  |  | 1289 | |  |
| 120 | M | 36 | 20 | M | E |  |  |  |  |  |  |  |  |  |  |  |  |  |  |  | 531 | |  |
| 121 | F | 47 | 37 | O | L |  |  |  |  |  |  |  |  |  | + |  |  |  |  |  | 1651 | |  |
| 122 | F | 36 | 26 | S | L | + |  |  |  |  |  |  |  |  |  |  |  |  |  | + | 4118 | |  |
| 123 | M | 25 | 22 | O | L |  |  |  |  |  |  |  |  |  |  |  |  |  |  |  | 6567 | |  |
| 124 | F | 37 | 33 | O | L |  |  |  |  |  |  |  |  |  |  |  |  |  |  |  | 2325 | |  |
| 125 | M | 28 | 21 | O | L |  | + |  |  |  |  |  |  |  |  |  |  |  |  |  | 7256 | |  |
| 126 | M | 47 | 33 | S | L |  | + |  |  |  |  |  |  |  |  |  |  |  |  |  | 3000 | |  |
| 127 | M | 47 | 36 | S | L | + | + |  |  |  |  |  |  |  |  |  | + |  |  |  | 464 | |  |
| 128 | F | 5 | 2 | O | L | + | + |  |  |  | + |  |  |  |  |  |  |  |  |  | 330 | |  |
| 129 | M | 13 | 10 | M | L | + |  |  |  | + | + |  |  |  |  |  |  |  |  |  | 6465 | |  |
| 130 | F | 14 | 5 | M | U | + |  |  |  |  | + |  |  |  |  |  |  |  |  |  | 312 | |  |
| 131 | M | 45 | 42 | M | U |  |  |  |  |  |  |  |  |  |  |  |  |  |  |  | 2279 | |  |
| 132 | M | 22 | 17 | M | L |  |  |  |  |  | + |  |  |  |  |  |  |  |  |  | 3999 | |  |
| 133 | F | 29 | 9 | S | L | + |  |  |  | + |  |  |  |  |  |  |  |  |  |  | 35 | |  |
| 134 | F | 36 | 29 | S | L | + | + |  |  |  |  |  |  |  |  |  |  |  |  |  | 2629 | |  |
| 135 | F | 31 | 24 | M | L | + |  |  |  |  | + |  |  |  |  |  |  |  |  |  | 2686 | |  |
| 136 | F | 37 | 29 | O | L | + | + |  |  |  | + |  |  |  | + |  |  |  |  |  | 900 | |  |
| 137 | F | 29 | 21 | S | L | + |  |  |  |  |  | + |  |  | + |  |  |  |  |  | 4289 | |  |
| 138 | M | 24 | 14 | O | L | + |  |  |  |  |  | + |  |  | + |  |  |  |  |  | 4443 | |  |
| 139 | F | 33 | 31 | M | L | + |  |  |  |  | + | + |  |  |  |  | + |  |  |  | 3642 | |  |
| 140 | F | 19 | 17 | M | L |  |  |  |  | + |  |  |  |  |  |  |  |  |  |  | 13627 | |  |
| 141 | F | 10 | 4 | O | L | + |  |  |  | + |  |  |  |  |  |  |  |  |  |  | 11831 | |  |
| 142 | F | 28 | 25 | M | L | + |  |  |  |  |  |  |  |  |  |  |  |  |  |  | 3912 | |  |
| 143 | M | 48 | 36 | S | L |  |  |  |  |  |  |  |  |  |  |  | + |  |  |  | 1290 | |  |
| 144 | F | 46 | 41 | O | U | + |  |  | + |  |  |  |  |  |  |  |  |  |  |  | 136 | |  |
| 145 | M | 26 | 25 | M | E |  |  |  |  |  |  |  |  |  |  |  |  |  |  |  | 12085.8 | |  |
| 146 | M | 28 | 8 | O | L |  |  |  |  |  |  |  |  |  |  |  |  |  |  |  | 5552 | |  |
| 147 | F | 41 | 37 | S | L | + | + |  | + |  |  |  | + |  |  |  |  |  |  |  | 706 | |  |
| 148 | M | 25 | 16 | M | L | + |  |  |  |  |  |  |  |  |  |  |  |  |  |  | 5755 | |  |
| 149 | M | 24 | 12 | S | L | + | + |  |  |  |  |  |  |  |  |  |  |  |  |  | 14000 | |  |
| 150 | F | 34 | 24 | M | L | + |  |  |  |  |  |  |  |  |  |  |  |  |  |  | 6000 | |  |
| 151 | F | 30 | 27 | M | L |  |  |  |  |  |  |  |  |  |  |  |  |  |  |  | 10898 | |  |
| 152 | M | 29 | 9 | O | L | + |  |  |  | + | + |  |  |  |  |  |  |  |  |  | 360 | |  |
| 153 | F | 57 | 37 | S | L |  | + |  |  |  |  |  |  |  |  |  | + |  |  |  | 332 | |  |
| 154 | M | 13 | 13 | M | L |  |  |  |  |  |  |  |  |  |  |  |  |  |  |  | 3461 | |  |
| 155 | F | 45 | 43 | M | U |  |  |  |  |  |  | + |  |  |  |  |  |  |  |  | 128 | |  |
| 156 | M | 40 | 30 | S | L | + | + |  |  |  |  |  |  |  | + |  |  |  |  |  | 712 | |  |
| 157 | M | 16 | 14 | M | L | + |  |  |  |  |  | + |  |  |  |  |  |  |  |  | 20000 | |  |
| 158 | F | 4 | 2 | M | L |  |  |  |  |  | + |  |  |  |  |  |  |  |  |  | 1005 | |  |
| 159 | M | 39 | 35 | M | L |  |  |  |  |  |  |  |  |  |  |  |  |  |  |  | 698 | |  |
| 160 | M | 19 | 15 | M | L |  |  |  |  |  | + |  |  |  |  |  |  |  |  |  | 8184 | |  |
| 161 | F | 14 | 4 | M | L | + |  |  | + |  | + |  |  |  |  | + |  |  |  |  | 820 | |  |
| 162 | M | 27 | 12 | O | L |  |  |  |  |  |  |  |  |  |  |  |  |  |  |  | 1490 | |  |
| 163 | M | 23 | 17 | O | L |  |  |  |  |  |  |  |  |  |  |  |  |  |  |  | 2788 | |  |
| 164 | M | 7 | 3 | M | E | + |  |  |  |  |  |  |  |  |  |  |  |  |  |  | 8000 | |  |
| 165 | M | 28 | 18 | O | E | + |  |  |  | + | + |  |  |  |  |  |  |  |  |  | 1000 | |  |
| 166 | M | 24 | 17 | M | L |  |  |  |  | + |  |  |  |  |  |  |  |  |  |  | 4136 | |  |
| 167 | M | 27 | 24 | M | L |  |  |  |  | + |  |  |  |  |  |  |  |  |  |  | 3220 | |  |
| 168 | M | 35 | 15 | S | L |  |  |  |  | + |  |  |  |  | + |  |  |  |  |  | 1353 | |  |
| 169 | M | 37 | 32 | S | L | + | + |  |  |  |  |  | + | + |  |  | + |  |  |  | 526 | |  |
| 170 | M | 52 | 47 | M | L | + |  |  |  |  |  |  |  |  |  |  |  |  |  |  | 3245 | |  |
| 171 | F | 11 | 10 | S | L | + |  |  |  |  |  |  |  |  | + |  |  |  |  |  | 5355 | |  |
| 172 | F | 46 | 36 | O | E | + |  |  |  |  |  |  |  |  |  |  |  |  |  |  | 412 | |  |
| 173 | M | 38 | 37 | O | L | + |  |  |  |  |  |  |  |  |  |  |  |  |  |  | 388 | |  |
| 174 | F | 52 | 42 | S | U |  | + |  |  |  |  |  |  |  |  |  |  |  |  |  | 2270 | |  |
| 175 | M | 5 | 2 | S | E | + | + |  |  |  |  |  |  |  | + | + |  |  |  |  | 444 | |  |
| 176 | F | 5 | 2 | S | U | + | + |  | + |  | + |  |  |  | + |  |  |  |  |  | 900 | |  |
| 177 | F | 28 | 14 | M | L |  | + |  |  |  | + |  |  |  |  |  |  |  |  |  | 11051 | |  |
| 178 | F | 32 | 30 | O | L | + | + |  |  |  |  | + |  |  |  |  |  |  |  |  | 5000 | |  |
| 179 | M | 4 | 2 | M | L |  |  |  |  |  |  |  |  |  | + |  |  |  |  |  | 23131 | |  |
| 180 | M | 21 | 15 | M | L | + |  |  |  |  |  |  |  |  |  |  |  |  |  |  | 18000 | |  |

a: based on the Medical Research Council (MRC) grade of the weakest muscle group, M: mild (MRC grade ≥4/5), O: moderate (MRC grade 3-4/5), S: severe (MRC grade <3/5).

b: L: lower limbs more severe than upper limbs; E: lowers limbs equal to upper limbs; U: upper limbs more severe than lower limbs.

c: maximum serum CK level.
