# Supplementary material for: Mutational spectrum of Chinese LGMD patients by targeted next-generation sequencing
Source: PLoS One. 2017 Apr 12;12(4):e0175343. doi: 10.1371/journal.pone.0175343 (PMC5389788; doi:10.1371/journal.pone.0175343)
Supplement: S3 Table — (DOCX) [file pone.0175343.s003.docx]

**Table e-3 Myopathological features of 180 patients suspected of LGMD**

| No | Pathological diagnosis | Lobulated fiber | Rimmed Vacuole | Non-rimmed vacuole | Nemaline body | Multi-core | Mitochondrial dysfunction^a^ | Dystrophin^b^ | Dysferlin^b^ | Sarcoglycan^b^ | Inflammation related^c^ |
| --- | --- | --- | --- | --- | --- | --- | --- | --- | --- | --- | --- |
| 1 | Dystrophic | + |  |  |  |  |  |  |  |  |  |
| 2 | Dystrophic |  |  |  |  |  |  | + | + | + | + |
| 3 | Dystrophic |  |  |  |  |  |  |  |  |  |  |
| 4 | Myopathic |  | + |  |  |  | + |  |  |  |  |
| 5 | Dystrophic |  |  |  |  |  | + |  |  |  |  |
| 6 | Dystrophic |  |  |  |  |  |  | + |  | + | + |
| 7 | Dystrophic |  |  |  |  |  |  | + |  | + | + |
| 8 | Myopathic |  |  |  |  |  |  |  | + | + | + |
| 9 | Myopathic |  | + |  |  |  |  |  |  |  |  |
| 10 | Dystrophic |  |  |  |  |  |  | + |  | + | + |
| 11 | Myopathic |  |  |  |  |  |  |  |  |  |  |
| 12 | Dystrophic |  |  |  |  |  |  | + |  | + |  |
| 13 | Dystrophic | + |  |  |  |  |  |  |  | + | + |
| 14 | Dystrophic |  |  |  |  |  |  | + |  | + | + |
| 15 | Dystrophic |  |  |  |  |  |  |  |  |  |  |
| 16 | Myopathic |  |  | + |  |  |  |  | + |  | + |
| 17 | Dystrophic |  |  |  |  |  |  |  | + |  | + |
| 18 | Dystrophic |  |  |  |  |  | + |  | + |  | + |
| 19 | Myopathic |  | + |  |  |  |  |  |  |  |  |
| 20 | Myopathic |  |  |  |  |  |  |  | + |  | + |
| 21 | Dystrophic |  |  |  |  |  |  |  |  | + |  |
| 22 | Dystrophic | + |  | + |  |  |  |  | + |  |  |
| 23 | Dystrophic |  | + |  |  |  |  | + |  |  | + |
| 24 | Dystrophic | + |  |  |  |  | + | + |  |  | + |
| 25 | Dystrophic |  | + |  |  |  |  |  |  |  | + |
| 26 | Myopathic |  | + |  |  |  |  |  |  |  |  |
| 27 | Dystrophic |  |  |  |  |  |  |  |  |  | + |
| 28 | Dystrophic |  |  |  |  |  |  |  | + |  | + |
| 29 | Dystrophic | + |  |  |  |  | + |  | + |  | + |
| 30 | Dystrophic |  |  |  |  |  | + |  | + |  | + |
| 31 | Dystrophic |  |  |  |  |  |  | + |  | + | + |
| 32 | Dystrophic |  |  |  |  |  |  |  | + |  | + |
| 33 | Dystrophic |  |  |  |  |  |  |  | + |  | + |
| 34 | Myopathic |  |  |  |  |  |  |  |  |  |  |
| 35 | Dystrophic |  |  |  |  |  |  |  |  |  |  |
| 36 | Dystrophic | + | + |  |  |  | + |  | + |  | + |
| 37 | Dystrophic |  | + |  |  |  |  | + |  | + | + |
| 38 | Dystrophic | + |  |  |  |  | + |  | + |  |  |
| 39 | Dystrophic | + |  |  |  |  |  |  | + |  |  |
| 40 | Myopathic |  |  |  |  |  |  |  |  |  |  |
| 41 | Dystrophic |  |  | + |  |  |  |  | + |  | + |
| 42 | Dystrophic |  |  |  |  |  |  |  |  |  | + |
| 43 | Dystrophic |  |  |  |  |  |  |  |  |  |  |
| 44 | Myopathic |  |  |  |  |  |  |  |  |  |  |
| 45 | Dystrophic |  |  | + |  |  |  | + |  |  | + |
| 46 | Dystrophic | + | + |  |  |  | + | + |  | + | + |
| 47 | Dystrophic |  |  |  |  |  | + |  | + |  | + |
| 48 | Myopathic |  |  | + |  |  |  |  | + |  | + |
| 49 | Dystrophic | + |  |  |  |  |  |  |  |  |  |
| 50 | Dystrophic |  |  |  |  |  | + |  | + |  | + |
| 51 | Dystrophic |  |  |  |  |  |  |  | + |  | + |
| 52 | Myopathic |  |  |  | + | + |  |  |  |  |  |
| 53 | Dystrophic |  | + |  |  |  |  |  |  |  | + |
| 54 | Dystrophic |  |  | + |  |  |  |  |  |  | + |
| 55 | Dystrophic |  | + |  |  |  |  |  |  |  | + |
| 56 | Dystrophic | + |  |  |  |  |  |  | + |  | + |
| 57 | Dystrophic |  |  |  |  |  |  | + |  |  | + |
| 58 | Dystrophic |  | + |  |  |  |  | + |  |  |  |
| 59 | Dystrophic |  |  |  |  |  |  | + |  |  |  |
| 60 | Myopathic |  |  |  |  |  |  |  |  |  |  |
| 61 | Myopathic |  |  |  |  |  |  |  |  |  |  |
| 62 | Dystrophic |  |  |  |  |  |  | + |  | + | + |
| 63 | Dystrophic |  |  |  |  |  | + | + |  | + | + |
| 64 | Myopathic |  |  | + |  |  |  |  | + |  |  |
| 65 | Myopathic | + | + |  |  |  |  |  |  |  |  |
| 66 | Dystrophic | + |  |  |  |  |  |  |  |  | + |
| 67 | Dystrophic |  |  |  |  |  |  |  |  |  | + |
| 68 | Myopathic |  |  |  |  |  |  |  |  |  |  |
| 69 | Dystrophic |  |  |  |  | + | + | + |  |  |  |
| 70 | Myopathic |  |  |  | + | + |  | + |  | + |  |
| 71 | Myopathic |  |  |  |  |  |  |  | + |  |  |
| 72 | Dystrophic |  |  |  |  |  |  |  |  |  | + |
| 73 | Dystrophic | + |  |  |  |  | + |  |  |  | + |
| 74 | Myopathic |  |  |  |  |  |  |  | + |  | + |
| 75 | Dystrophic |  |  |  |  |  |  | + |  | + |  |
| 76 | Myopathic |  |  | + |  |  |  |  |  |  |  |
| 77 | Dystrophic |  |  |  |  |  | + |  | + |  | + |
| 78 | Dystrophic |  |  |  |  |  |  |  |  | + |  |
| 79 | Myopathic |  |  |  |  |  |  |  |  |  |  |
| 80 | Dystrophic |  |  |  |  |  |  |  | + |  | + |
| 81 | Myopathic |  |  |  |  |  |  |  |  |  | + |
| 82 | Dystrophic |  |  |  |  |  |  |  |  |  | + |
| 83 | Dystrophic | + |  |  |  |  | + |  | + |  | + |
| 84 | Dystrophic | + | + |  |  |  |  |  |  |  |  |
| 85 | Myopathic |  |  |  |  |  |  |  |  |  | + |
| 86 | Myopathic |  | + |  |  |  | + |  |  |  | + |
| 87 | Dystrophic |  |  |  |  |  | + |  | + |  | + |
| 88 | Dystrophic |  |  |  |  |  |  |  |  |  |  |
| 89 | Dystrophic |  |  |  |  |  |  | + |  | + | + |
| 90 | Dystrophic |  |  |  |  |  |  | + |  | + | + |
| 91 | Myopathic |  | + |  |  |  | + |  |  |  |  |
| 92 | Myopathic |  |  |  |  |  |  |  |  |  | + |
| 93 | Myopathic |  |  |  |  |  |  |  |  |  |  |
| 94 | Dystrophic | + |  |  |  |  |  |  |  |  |  |
| 95 | Dystrophic |  |  |  |  |  |  |  |  |  | + |
| 96 | Dystrophic | + |  |  |  |  | + | + | + |  | + |
| 97 | Myopathic |  |  |  |  | + |  |  |  |  | + |
| 98 | Dystrophic |  |  |  |  |  |  |  |  |  | + |
| 99 | Myopathic |  |  |  |  |  |  |  | + |  | + |
| 100 | Dystrophic |  |  |  |  |  |  |  |  |  | + |
| 101 | Myopathic |  |  |  |  |  |  |  |  |  | + |
| 102 | Dystrophic |  |  |  |  |  |  |  |  | + |  |
| 103 | Myopathic |  |  | + |  |  |  |  | + |  | + |
| 104 | Dystrophic |  |  |  |  |  |  | + |  | + |  |
| 105 | Myopathic |  |  | + |  |  |  | + |  |  |  |
| 106 | Myopathic |  | + |  |  |  |  |  |  |  |  |
| 107 | Myopathic |  |  |  |  | + |  |  |  |  |  |
| 108 | Dystrophic |  | + |  |  |  |  | + |  | + |  |
| 109 | Dystrophic |  |  |  |  |  |  | + |  | + | + |
| 110 | Dystrophic |  |  |  |  |  |  |  |  |  |  |
| 111 | Dystrophic |  |  |  |  |  |  | + |  | + | + |
| 112 | Myopathic |  | + |  |  |  |  |  |  |  | + |
| 113 | Dystrophic |  |  |  |  |  |  |  | + | + | + |
| 114 | Dystrophic |  |  |  |  |  |  |  |  | + |  |
| 115 | Dystrophic |  |  |  |  |  |  | + | + | + | + |
| 116 | Dystrophic |  |  |  |  |  |  | + |  | + | + |
| 117 | Myopathic |  |  |  |  |  |  |  |  |  | + |
| 118 | Dystrophic |  |  |  |  |  |  |  | + |  | + |
| 119 | Dystrophic | + |  |  |  |  |  |  |  |  |  |
| 120 | Dystrophic |  |  |  |  |  |  |  |  |  |  |
| 121 | Myopathic |  |  |  |  |  |  |  |  |  |  |
| 122 | Dystrophic |  |  |  |  |  | + |  | + |  | + |
| 123 | Myopathic |  |  |  |  |  |  |  | + |  | + |
| 124 | Dystrophic | + |  |  | + |  |  |  | + |  |  |
| 125 | Dystrophic |  | + |  |  |  | + |  | + |  | + |
| 126 | Myopathic |  |  | + |  |  |  | + |  | + | + |
| 127 | Myopathic |  | + |  |  |  |  |  |  |  | + |
| 128 | Dystrophic |  |  |  |  |  |  |  |  |  | + |
| 129 | Dystrophic |  |  |  |  |  |  | + |  | + | + |
| 130 | Dystrophic | + |  |  |  |  |  | + |  | + |  |
| 131 | Dystrophic | + | + |  |  |  |  | + | + | + | + |
| 132 | Dystrophic |  |  |  |  |  |  | + |  |  | + |
| 133 | Myopathic |  |  |  |  | + |  |  |  |  |  |
| 134 | Dystrophic |  | + |  |  |  | + |  | + |  | + |
| 135 | Dystrophic |  | + |  |  |  |  | + | + |  | + |
| 136 | Dystrophic | + |  |  |  |  |  |  |  |  | + |
| 137 | Dystrophic |  |  |  |  |  |  | + |  | + | + |
| 138 | Dystrophic | + |  |  |  |  |  | + |  | + | + |
| 139 | Dystrophic |  |  | + |  |  | + |  | + |  | + |
| 140 | Dystrophic |  |  |  |  |  |  |  | + |  | + |
| 141 | Dystrophic |  |  |  |  |  |  | + |  | + | + |
| 142 | Dystrophic |  |  |  |  |  | + | + | + |  | + |
| 143 | Myopathic |  | + |  |  |  |  |  |  |  |  |
| 144 | Dystrophic | + |  |  |  |  |  |  |  |  | + |
| 145 | Dystrophic |  |  | + |  |  |  |  | + |  | + |
| 146 | Dystrophic |  |  |  |  |  | + | + |  | + |  |
| 147 | Dystrophic |  |  |  |  |  |  |  |  |  |  |
| 148 | Dystrophic |  |  |  |  |  | + |  | + |  | + |
| 149 | Dystrophic |  |  |  |  |  |  | + | + |  | + |
| 150 | Dystrophic |  |  |  |  |  |  | + | + |  | + |
| 151 | Myopathic |  |  |  |  |  | + |  | + |  | + |
| 152 | Myopathic |  |  |  |  | + |  |  |  |  | + |
| 153 | Myopathic |  |  |  |  | + |  |  |  |  | + |
| 154 | Dystrophic |  |  |  |  |  |  |  | + |  | + |
| 155 | Myopathic | + | + |  |  |  |  |  |  |  |  |
| 156 | Myopathic |  | + |  |  |  |  |  |  |  |  |
| 157 | Dystrophic |  |  |  |  |  |  |  | + |  | + |
| 158 | Dystrophic |  |  |  |  |  |  | + |  |  | + |
| 159 | Dystrophic |  |  | + |  |  |  |  |  |  |  |
| 160 | Dystrophic |  |  |  |  |  |  |  |  |  | + |
| 161 | Dystrophic |  |  |  |  |  |  |  |  |  | + |
| 162 | Dystrophic |  |  |  |  |  |  | + | + |  | + |
| 163 | Dystrophic |  |  |  |  |  |  |  |  |  | + |
| 164 | Dystrophic |  |  |  |  |  |  | + |  | + |  |
| 165 | Dystrophic |  |  |  |  |  |  | + |  | + | + |
| 166 | Dystrophic |  |  |  |  |  |  | + |  | + |  |
| 167 | Dystrophic |  |  |  |  |  |  | + |  | + |  |
| 168 | Dystrophic | + |  |  |  |  |  |  |  |  |  |
| 169 | Myopathic |  |  |  |  |  |  |  |  |  | + |
| 170 | Dystrophic | + |  |  |  |  |  | + |  |  |  |
| 171 | Dystrophic |  |  |  |  |  |  | + |  | + | + |
| 172 | Dystrophic | + | + |  |  |  |  | + |  |  | + |
| 173 | Dystrophic |  |  |  |  |  |  | + |  | + | + |
| 174 | Dystrophic |  |  |  |  |  |  | + |  | + | + |
| 175 | Dystrophic |  |  |  |  |  |  | + |  | + | + |
| 176 | Dystrophic |  |  |  |  |  |  |  |  |  | + |
| 177 | Dystrophic |  |  |  |  |  | + |  | + |  | + |
| 178 | Myopathic |  |  |  |  |  |  |  | + |  | + |
| 179 | Dystrophic |  |  |  |  |  |  |  |  | + |  |
| 180 | Myopathic |  |  |  |  |  |  |  | + |  | + |

a: including RRF, RBF and COX-negative fibers.

b: absent or decreased expression.

c: including expression of major histocompatibility complex-I (MHC-I) and deposit of complement (C5b-9).
