# Supplementary material for: Mutational spectrum of Chinese LGMD patients by targeted next-generation sequencing
Source: PLoS One. 2017 Apr 12;12(4):e0175343. doi: 10.1371/journal.pone.0175343 (PMC5389788; doi:10.1371/journal.pone.0175343)
Supplement: S4 Table — (DOCX) [file pone.0175343.s004.docx]

**Table e-4 Variants detected by targeted NGS in 180 patients suspected of LGMD**

| No | Gene | Base change | AA change | Parental Origin | Zygosity |
| --- | --- | --- | --- | --- | --- |
| 1 | *CAPN3* | c.1485delA | p.A497Pfs*98 |  | hom |
| 2 | *DYSF* | c.4513 T>A | p.Y1505N |  | het |
| 3 | *ITGA7* | c.2701A>G | p.I901V |  | het |
|  |  | c.1828G>A | p.G610R |  | het |
| 4 | *-* | - | - |  | - |
| 5 | *-* | - | - |  | - |
| 6 | *-* | - | - |  | - |
| 7 | *CAPN3* | c.1333G>A | p.G445R |  | het |
|  |  | c.2305C>T | p.R769W |  | het |
| 8 | *DYSF* | c.1464delT | p.G489Efs*4 |  | het |
| 9 | *GNE* | c.910G>A | p.G304R | Paternal | het |
|  |  | c.527A>T | p.D176V | Maternal | het |
| 10 | *-* | - | - |  | - |
| 11 | *CAPN3* | c.1194-9A>G | splicing |  | het |
|  |  | c.2120A>G | p.D707G |  | het |
| 12 | *LMNA* | c.1357C>T | p.R453W | De novo | het |
| 13 | *SGCA* | c.662G>A | p.R221H |  | het |
| 14 | - | - | - |  | - |
| 15 | *CAPN3* | c.691dupT | p.T232Hfs*9 |  | hom |
| 16 | *KBTBD13* | c.252C>A | p.C84Ter |  | het |
| 17 | *DYSF* | c.4509+2_c.4509+6delTAAGG | splicing |  | hom |
| 18 | *DYSF* | c.308dupC | p.L105Pfs*43 | Paternal | het |
|  |  | c.5438T>C | p.L1813P | Maternal | het |
| 19 | *-* | - | - |  | - |
| 20 | *DYSF* | c.3601C>T | p.Q1201Ter | Paternal | het |
|  |  | c.1180+5G>A | splicing | Maternal | het |
| 21 | *SGCA* | c.95T>C | p.V32A | Paternal | het |
|  |  | c.313-2A>G | splicing | Maternal | het |
| 22 | *DYSF* | c.956A>T | p.D319V | Paternal | het |
|  |  | c.1058T>C | p.L353P | Maternal | het |
| 23 | *DES* | c.155G>T | p.R52L | De novo | het |
| 24 | *CAPN3* | c.25C>T | p.Q9Ter |  | het |
|  |  | c.264+1G>C | splicing |  | het |
| 25 | - | - | - |  | - |
| 26 | - | - | - |  | - |
| 27 | *MYOT* | c.1318G>A | p.V440I |  | het |
| 28 | *DYSF* | c.2940delG | p.L981Ffs*76 | Paternal | het |
|  |  | c.4194dupC | p.I1401Hfs*8 | Maternal | het |
| 29 | *DYSF* | c.252delC | p.K85Rfs*66 |  | hom |
| 30 | *DYSF* | c.1992C>A | p.W664Ter | Paternal | het |
|  |  | c.2997G>T | p.W999C | Maternal | het |
| 31 | *DMD* | c.118delC | p.L40Sfs*12 |  | het |
| 32 | *DYSF* | c.799_800delTT | p.F267Lfs*5 |  | hom |
| 33 | *DYSF* | c.937+1G>A | splicing | Paternal | het |
|  |  | c.3521-1G>T | splicing | Maternal | het |
| 34 | *-* | - | - |  | - |
| 35 | - | - | - |  | - |
| 36 | *DYSF* | c.2082delG | p.A695Pfs*2 |  | hom |
| 37 | *-* | - | - |  | - |
| 38 | *DYSF* | c.176delT | p.L59Rfs*92 | Paternal | het |
|  |  | c.5975delT | p.V1992Efs*20 | Maternal | het |
| 39 | *DYSF* | c.4194delC | p.I1401Sfs*47 | Paternal | het |
|  |  | c.4886+2T>G | splicing | Maternal | het |
| 40 | *-* | - | - |  | - |
| 41 | *DYSF* | c.610C>T | p.R204Ter | Paternal | het |
|  |  | c.3516_3517delTT | p.S1173Ter | Maternal | het |
| 42 | *COL6A1* | c.2737G>A | p.A913T |  | het |
| 43 | *FKRP* | c.545A>G | p.Y182C | Paternal | het |
|  |  | c.1067T>C | p.I356T | Maternal | het |
| 44 | - | - | - |  | - |
| 45 | *-* | - | - |  | - |
| 46 | *CAPN3* | c.2120A>G | p.D707G | Paternal | het |
|  |  | c.2243G>A | p.R748Q | Maternal | het |
| 47 | *DYSF* | c.863A>T | p.D288V | Paternal | het |
|  |  | c.5077C>T | p.R1693W | Maternal | het |
| 48 | *DYSF* | c.3032-3C>G | splicing | Paternal | het |
|  |  | c.5639C>G | p.A1880G | Maternal | het |
| 49 | *LMNA* | c.1152G>C | p.E384D | De novo | het |
| 50 | *DYSF* | c.1667T>C | p.L556P |  | hom |
| 51 | *DYSF* | c.1180+5G>A | splicing |  | hom |
| 52 | *FLNC* | c.3706C>T | p.P1236S |  | het |
| 53 | *COL6A2* | c.1745G>A | p.G582D | De novo | het |
| 54 | *-* | - | - |  | - |
| 55 | *LMNA* | c.695G>A | p.G232E | De novo | het |
| 56 | *DYSF* | c.3516_3517delTT | p.S1173Ter |  | hom |
| 57 | *CAPN3* | c.733dupC | p.S246Ter | Paternal | het |
|  |  | c.1343G>T | p.R448L | Maternal | het |
| 58 | *DMD* | c.264delT | p.N88fs |  | hem |
| 59 | *DMD* | c.1704+1G>A | splicing |  | hem |
| 60 | - | - | - |  | - |
| 61 | *-* | - | - |  | - |
| 62 | *CAPN3* | c.734dupC | p.S246Ter |  | het |
|  |  | c.2243G>A | p.R748Q |  | het |
| 63 | *SGCB* | c.334C>T | p.Q112Ter |  | het |
| 64 | *DYSF* | c.3980C>T | p.P1327L |  | het |
|  |  | c.4985C>T | p.T1662M |  | het |
| 65 | *GNE* | c.38G>C | p.C13S |  | hom |
| 66 | *COL6A1* | c.842G>A | p.G281E |  | het |
| 67 | *LMNA* | c.1124C>G | p.A375G | De novo | het |
| 68 | *-* | - | - |  | - |
| 69 | *LAMA2* | c.1084_1085insTT | p.R362Ifs*4 | De novo | het |
| 70 | *CAPN3* | c.727insG | p.D243Gfs*4 |  | het |
|  |  | c.2081_2082delTG | p.L694Rfs*5 |  | het |
| 71 | *DYSF* | c.5444G>T | p.C1815F |  | hom |
| 72 | *-* | - | - |  | - |
| 73 | *-* | - | - |  | - |
| 74 | *DYSF* | c.1375dupA | p.M459Nfs*15 |  | het |
|  |  | c.5197A>G | p.I1733V |  | het |
| 75 | *-* | - | - |  | - |
| 76 | *PLEC* | c.9785T>A | p.F3262Y |  | het |
|  |  | c.6971G>A | p.R2324Q |  | het |
| 77 | *DYSF* | c.1523-2A>G | splicing |  | hom |
| 78 | *SGCA* | c.424A>G | p.S142G |  | het |
| 79 | *NEB* | c.14071C>G | p.H4691D | Maternal | het |
|  |  | c.3352G>T | p.A1118S | De novo | het |
| 80 | *DYSF* | c.796_797delCT | p.L266Ffs*6 | Paternal | het |
|  |  | c.1377_1379delGAG | p.R460del | Maternal | het |
| 81 | *FKRP* | c.545A>G | p.Y182C |  | hom |
|  |  | c.1027G>C | p.E343Q |  | het |
| 82 | *CAPN3* | c.255_256delCT | p.F88Lfs*2 | Paternal | het |
|  |  | c.1900A>T | p.K634Ter | Maternal | het |
| 83 | *DYSF* | c.3115C>T | p.R1039W | Paternal | het |
|  |  | c.5245C>T | p.R1749C | Maternal | het |
|  |  | c.5525G>A | p.G1842D | Maternal | het |
| 84 | *FLNC* | c.5278G>A | p.G1760S |  | het |
| 85 | *-* | - | - |  | - |
| 86 | *DES* | c.1109T>C | p.L370P | De novo | het |
| 87 | *DYSF* | c.1375dupA | p.M459Nfs*15 | Paternal | het |
|  |  | c.2030-?_2439+?del |  | De novo | het |
| 88 | *CAPN3* | c.2120A>G | p.D707G |  | het |
| 89 | *SGCA* | c.292C>T | p.R98C | Paternal | het |
|  |  | c.889delC | p.L298Cfs*23 | Maternal | het |
| 90 | *DMD* | c.1704+1G>T | splicing |  | hem |
| 91 | *-* | - | - |  | - |
| 92 | *LAMA2* | c.910-1G>T | splicing |  | het |
| 93 | *IGHMBP2* | c.2960G>A | p.R987Q |  | het |
| 94 | *TTN* | c.87877C>T | p.R29293C |  | het |
|  |  | c.80115G>T | p.E26705D |  | het |
|  |  | c.60850A>G | p.T20284A |  | het |
|  |  | c.187G>A | p.A63T |  | het |
| 95 | *CAPN3* | c.1561C>T | p.Q521Ter |  | het |
|  |  | c.2289T>G | p.Y763Ter |  | het |
| 96 | *DYSF* | c.895G>C | p.G299R | Paternal | het |
|  |  | c.4756C>T | p.R1586Ter | Maternal | het |
| 97 | *RYR1* | c.7804G>A | p.V2602I |  | het |
| 98 | *-* | - | - |  | - |
| 99 | *DYSF* | c.1874A>T | p.D625V | Paternal | het |
|  |  | c.2762C>T | p.S921L | Paternal | het |
|  |  | c.3785delG | p.G1263Afs*82 | Maternal | het |
| 100 | *POMT1* | c.260dupT | p.L87Ffs*11 |  | het |
|  |  | c.1417T>C | p.S473P |  | het |
| 101 | *-* | - | - |  | - |
| 102 | *SGCA* | c.95T>C | p.V32A | Paternal | het |
|  |  | c.409G>C | p.E137Q | Maternal | het |
| 103 | *DYSF* | c.1535_1553del | p.F514Pfs*107 | Paternal | het |
|  |  | c.4167+1G>A | splicing | Maternal | het |
| 104 | *DMD* | c.3432+3A>G | splicing |  | hem |
| 105 | *-* | - | - |  | - |
| 106 | *-* | - | - |  | - |
| 107 | *-* | - | - |  | - |
| 108 | *DMD* | c.513T>A | p.H171Q |  | hem |
| 109 | *-* | - | - |  | - |
| 110 | *-* | - | - |  | - |
| 111 | *DMD* | c.7224dupT | p.E2409Ter |  | het |
| 112 | *-* | - | - |  | - |
| 113 | *DYSF* | c.4988_4989delTC | p.V1663Gfs*47 |  | hom |
| 114 | *SGCA* | c.320C>T | p.A107V | Paternal | het |
|  |  | c.661C>T | p.R221C | Maternal | het |
| 115 | *DYSF* | c.1523-2A>G | splicing | Paternal | het |
|  |  | c.2974T>C | p.W992R | Maternal | het |
| 116 | *-* | - | - |  | - |
| 117 | *CAPN3* | c.1309C>G | p.R437G |  | het |
|  |  | c.2065_2066delAC | p.H690Rfs*9 |  | het |
| 118 | *DYSF* | c.1180+5G>A | splicing | Paternal | het |
|  |  | c.4194delC | p.I1401Sfs*47 | Maternal | het |
| 119 | *CAPN3* | c.802-9G>A | splicing |  | hom |
| 120 | *-* | - | - |  | - |
| 121 | *CAPN3* | c.2092C>T | p.R698C |  | het |
|  |  | c.2120A>G | p.D707G |  | het |
| 122 | *DYSF* | c.3601C>T | p.Q1201Ter | Paternal | het |
|  |  | c.4580T>G | p.L1527R | Maternal | het |
| 123 | *DYSF* | c.1930+2T>G | splicing |  | hom |
| 124 | *CAPN3* | c.1312delT | p.W438Gfs*25 |  | hom |
|  |  | c.2120A>G | p.D707G |  | het |
| 125 | *DYSF* | c.5884C>T | p.Q1962Ter |  | hom |
| 126 | *COL6A1* | c.1576-1G>T | splicing | De novo | het |
| 127 | *DES* | c.1030T>C | p.S344P | De novo | het |
| 128 | *CHRNA1* | c.1408G>A | p.V470M |  | het |
| 129 | *SGCD* | c.712-?_813+?dup |  |  | het |
| 130 | *-* | - | - |  | - |
| 131 | *DYSF* | c.3112C>T | p.R1038Ter |  | het |
| 132 | *CAPN3* | c.1621C>T | p.R541W | Paternal | het |
|  |  | c.2305C>T | p.R769W | Maternal | het |
| 133 | *MYH7* | c.4522_4524delGAG | p.E1507del |  | het |
| 134 | *DYSF* | c.3531_3533delCAT | p.1177_1178del |  | hom |
|  |  | c.5355G>A | p.M1785I |  | hom |
| 135 | *DYSF* | c.3702T>G | p.Y1234Ter | Paternal | het |
|  |  | c.5511C>A | p.D1837E | Maternal | het |
|  |  | c.5516A>T | p.Y1839F | Maternal | het |
| 136 | *CAPN3* | c.362T>A | p.I121N | Paternal | het |
|  |  | c.2116-1G>A | splicing | Maternal | het |
| 137 | *CAPN3* | c.1309C>G | p.R437G | Paternal | het |
|  |  | c.2289T>G | p.Y763Ter | Maternal | het |
| 138 | *CAPN3* | c.1413-9A>G | splicing | De novo | het |
|  |  | c.55+1G>A | splicing | Paternal | het |
| 139 | *DYSF* | c.707A>G | p.N236S | Paternal | het |
|  |  | c.4024C>G | p.R1342G | Paternal | het |
|  |  | c.4096G>A | p.V1366M | Paternal | het |
|  |  | c.5618-?_5706+?del |  | Maternal | het |
| 140 | *DYSF* | c.3258_3259delAG | p.D1087Cfs*26 |  | hom |
| 141 | *-* | - | - |  | - |
| 142 | *DYSF* | c.4022T>C | p.L1341P |  | hom |
| 143 | *-* | - | - |  | - |
| 144 | *CAPN3* | c.945+5G>A | splicing |  | het |
| 145 | *DYSF* | c.265C>T | p.R89Ter | Paternal | het |
|  |  | c.5509G>A | p.D1837N | Maternal | het |
| 146 | *DMD* | c.625G>A | p.D209N |  | hem |
| 147 | *CLCN1* | c.1582+2T>C | splicing |  | het |
| 148 | *DYSF* | c.1284+2T>C | splicing | Paternal | het |
|  |  | c.2254C>A | p.H752N | Maternal | het |
|  |  | c.4756C>T | p.R1586Ter | Maternal | het |
| 149 | *DYSF* | c.144+1G>A | splicing | Paternal | het |
|  |  | c.1393G>C | p.D465H | Maternal | het |
| 150 | *DYSF* | c.863A>T | p.D288V |  | hom |
| 151 | *DYSF* | c.1375dupA | p.M459Nfs*15 | Paternal | het |
|  |  | c.3036G>C | p.W1012C | Maternal | het |
| 152 | *COL6A2* | c.11_23delGCACCTGCTCCGT | p.T5Sfs*62 |  | het |
|  |  | c.1798G>A | p.E600K |  | het |
| 153 | *CAPN3* | c.145C>T | p.R49C | Paternal | het |
|  |  | c.2120A>G | p.D707G | Maternal | het |
| 154 | *DYSF* | c.2643+5G>C | splicing |  | het |
|  |  | c.3827T>C | p.L1276P |  | het |
| 155 | *CLCN1* | c.313C>T | p.R105C |  | het |
| 156 | *GNE* | c.350A>T | p.D117V |  | hom |
| 157 | *DYSF* | c.3059C>T | p.P1020L | Paternal | het |
|  |  | c.3442+1G>A; | splicing | Paternal | het |
|  |  | c.6029A>C | p.N2010T | Maternal | het |
| 158 | *LMNA* | c.1357C>T | p.R453W |  | het |
| 159 | *TRIM32* | c.1121C>T | p.P374L |  | hom |
| 160 | *CAPN3* | c.1064G>C | p.R355P | Paternal | het |
|  |  | c.2120A>G | p.D707G | Maternal | het |
| 161 | *LMNA* | c.1357C>T | p.R453W |  | het |
| 162 | *CAPN3* | c.204T>A | p.C68Ter |  | hom |
| 163 | *CAPN3* | c.638A>G | p.H213R | Paternal | het |
|  |  | c.1561C>T | p.Q521Ter | Maternal | het |
| 164 | *SGCA* | c.233_234insGA | p.Y78Ter | Paternal | het |
|  |  | c.371T>C | p.I124T | Maternal | het |
| 165 | *DMD* | c.357+1G>T | splicing |  | hem |
| 166 | *-* | - | - |  | - |
| 167 | *-* | - | - |  | - |
| 168 | *-* | - | - |  | - |
| 169 | *DES* | c.821T>C | p.L274P | De novo | het |
| 170 | *ANO5* | c.148C>T | p.R50Ter | Paternal | het |
|  |  | c.1898+1G>A | splicing | Maternal | het |
| 171 | *SGCA* | c.229C>T | p.R77C |  | het |
| 172 | *CAPN3* | c.1093A>T | p.I365F | Paternal | het |
|  |  | c.1829C>T | p.A610V | Maternal | het |
| 173 | *-* | - | - |  | - |
| 174 | *PLEC* | c.3092T>G | p.V1031G |  | het |
| 175 | *LMNA* | c.736G>A | p.E246K |  | het |
| 176 | *-* | - | - |  | - |
| 177 | *DYSF* | c.5302C>T | p.R1768W |  | hom |
| 178 | *DYSF* | c.2997G>T | p.W999C |  | het |
|  |  | c.5639C>G | p.A1880G |  | het |
| 179 | *FKRP* | c.545A>G | p.Y182C |  | het |
|  |  | c.1263C>A | p.Y421Ter |  | het |
| 180 | *DYSF* | c.1667T>C | p.L556P | Paternal | het |
|  |  | c.4876G>A | p.V1626I | Paternal | het |
|  |  | c.3988C>T | p.Q1330Ter | Maternal | het |

Abbreviations: het=heterozygous, hom=homozygous, hem=hemizygous.
