# Supplementary material for: Mutational spectrum of Chinese LGMD patients by targeted next-generation sequencing
Source: PLoS One. 2017 Apr 12;12(4):e0175343. doi: 10.1371/journal.pone.0175343 (PMC5389788; doi:10.1371/journal.pone.0175343)
Supplement: S5 Table — (DOCX) [file pone.0175343.s005.docx]

**Table e-5 Clinical and pathological features of 15 patients with variants of uncertain significance**

| No | Sex | Onset age | Clinical features | CK (IU/L) | Pathological findings | Gene | Base change | AA change |  |  |
| --- | --- | --- | --- | --- | --- | --- | --- | --- | --- | --- |
| 3 | F | 2 | Proximal muscle weakness with facial weakness and dropping head | 160 | Dystrophic | *ITGA7* | c.2701A>G | p.I901V |  |  |
|  |  |  |  |  |  |  | c.1828G>A | p.G610R |  |  |
| 16 | F | 17 | Proximal lower limb weakness with paroxysmal cramps | 5065.2 | Myopathic with vacuoles | *KBTBD13* | c.252C>A | p.C84Ter |  |  |
| 27 | M | 3 | Proximal muscle weakness with ankle contracture | 213 | Dystrophic | *MYOT* | c.1318G>A | p.V440I |  |  |
| 42 | M | 2 | Proximal muscle weakness with ankle contracture, father and father's sister share a milder phenotype | 653 | Dystrophic | *COL6A1* | c.2737G>A | p.A913T |  |  |
| 52 | F | 12 | Proximal muscle weakness with wrist overflexion and Gothic arch | 34 | Myopathic with nemaline bodies | *FLNC* | c.3706C>T | p.P1236S |  |  |
| 66 | F | 13 | Proximal muscle weakness, son shares a more serious phenotype with onest since birth | 216 | Dystrophic | *COL6A1* | c.842G>A | p.G281E |  |  |
| 76 | M | 3 | Proximal muscle weakness with fatigue | 5371 | Myopathic with vacuoles | *PLEC* | c.9785T>A | p.F3262Y |  |  |
|  |  |  |  |  |  |  | c.6971G>A | p.R2324Q |  |  |
| 79 | M | 19 | Proximal lower limb weakness | 214 | Myopathic | *NEB* | c.14071C>G | p.H4691D |  |  |
|  |  |  |  |  |  |  | c.3352G>T | p.A1118S |  |  |
| 84 | M | 43 | Proximal muscle weakness | 487 | Dystrophic with rimmed vacuoles | *FLNC* | c.5278G>A | p.G1760S |  |  |
| 94 | M | 26 | Proximal muscle weakness | 123 | Dystrophic with lobulated fibers | *TTN* | c.87877C>T | p.R29293C |  |  |
|  |  |  |  |  |  |  | c.80115G>T | p.E26705D |  |  |
|  |  |  |  |  |  |  | c.60850A>G | p.T20284A |  |  |
|  |  |  |  |  |  |  | c.187G>A | p.A63T |  |  |
| 97 | F | 43 | Proximal muscle weakness, both daughters share similar phenotypes | 214 | Myopathic with multi-minicores | *RYR1* | c.7804G>A | p.V2602I |  |  |
| 128 | F | 2 | Proximal muscle weakness | 330 | Dystrophic | *CHRNA1* | c.1408G>A | p.V470M |  |  |
| 133 | F | 9 | Proximal muscle weakness | 35 | Myopathic with multi-minicores | *MYH7* | c.4522_4524delGAG | p.E1507del |  |  |
| 155 | F | 43 | Proximal lower limb weakness with paroxysmal cramps | 128 | Myopathic with rimmed vacuoles | *CLCN1* | c.313C>T | p.R105C |  |  |
| 174 | F | 42 | Proximal muscle weakness | 2270 | Dystrophic | *PLEC* | c.3092T>G | p.V1031G |  |  |
