# Supplementary figures and images for: Mutational spectrum of Chinese LGMD patients by targeted next-generation sequencing
Source: PLoS One. 2017 Apr 12;12(4):e0175343. doi: 10.1371/journal.pone.0175343 (PMC5389788; doi:10.1371/journal.pone.0175343)

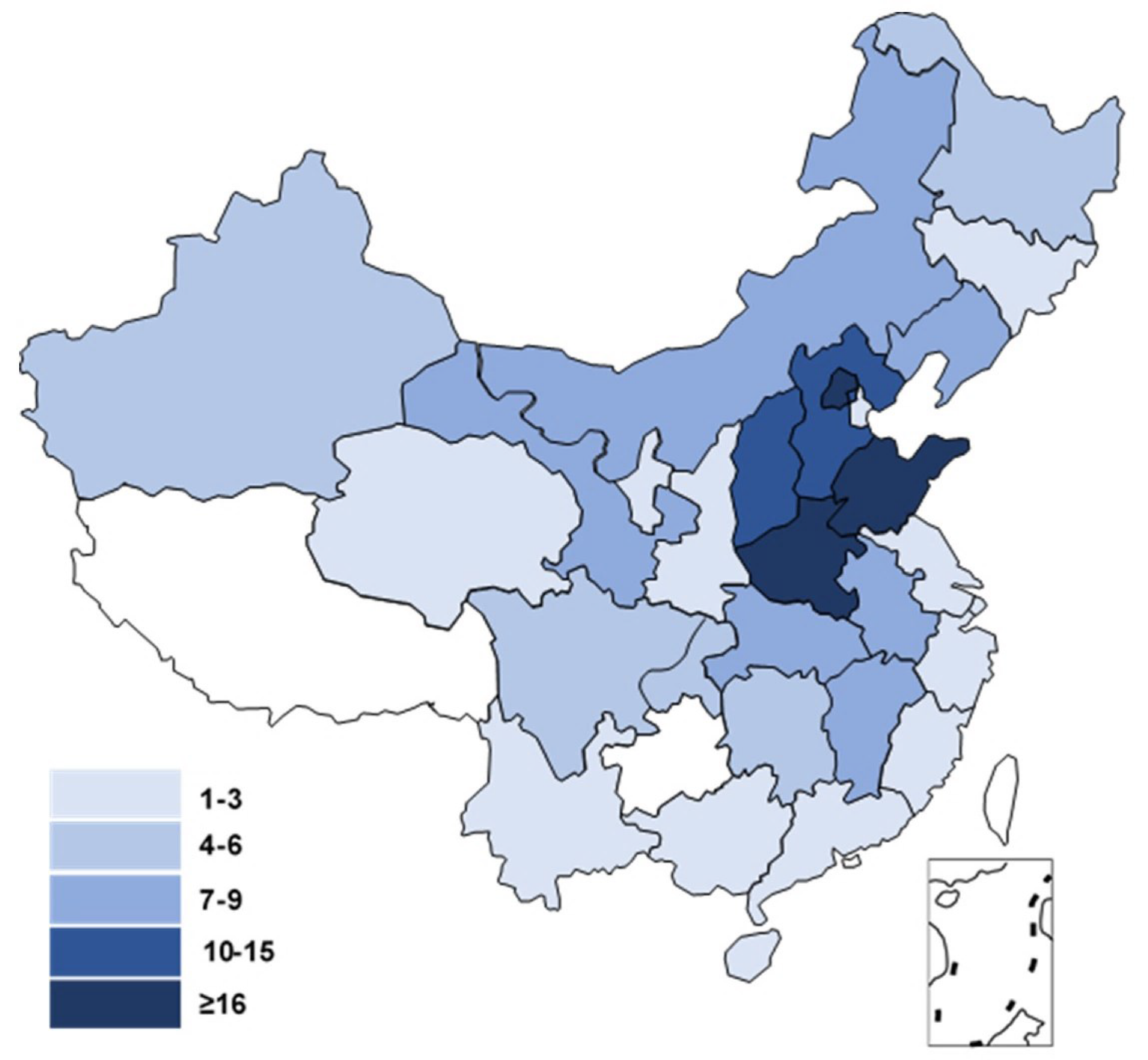

Supplement: S1 Fig — (TIFF) [file pone.0175343.s006.tiff]
